# Multimodality Fusion with MRI, CT, and Ultrasound Contrast for Ablation of Renal Cell Carcinoma

**DOI:** 10.1155/2012/390912

**Published:** 2012-12-13

**Authors:** Hayet Amalou, Bradford J. Wood

**Affiliations:** ^1^Center for Interventional Oncology, NIH Clinical Center, National Institutes of Health, MSC 1182, Bethesda, MD 20892, USA; ^2^Urologic Oncology Branch, National Cancer Institute, National Institutes of Health, Bethesda, MD 20892, USA

## Abstract

Fusion technology with electromagnetic (EM) tracking enables navigation with multimodality feedback that lets the operator use different modalities during different parts of the image-guided procedure. This may be particularly helpful in patients with renal insufficiency undergoing kidney tumor ablation, in whom there is a desire to minimize or avoid nephrotoxic iodinated contrast exposure. EM tracking software merges and fuses different imaging modalities such as MRI, CT, and ultrasound and can also display the position of needles in real time in relation to preprocedure imaging, which may better define tumor targets than available intraoperative imaging. EM tracking was successfully used to ablate a poorly visualized renal tumor, through the combined use of CT, gadolinium-enhanced MR, and contrast-enhanced US imaging to localize the tumor.

## 1. Introduction

Although surgical resection remains the gold standard for treating renal cell carcinoma, radiofrequency ablation (RFA) or cryoablation of renal lesions is widely becoming accepted as an effective treatment modality for patients that are poor surgical candidates, or in whom poor renal function necessitates a nephron-sparing approach [1]. The most technically challenging portion of an ablation procedure may be the localization of the tumor margins, which may be best defined on a modality (or enhancement phase) not immediately available during the procedure. Renal tumors may be poorly visualized with unenhanced CT or US and may be only visible on contrast-enhanced ultrasound (US), CT, and MRI [1]. However, MR-guided interventions are limited by cost, availability, and special equipment needs.

EM tracking allows real-time visualization of needle-tip position and angle of trajectory superimposed upon a pre-procedural image. Such systems require an electromagnetic field generator and a special introducer needle or stylet with a sensor coil embedded within an introducer needle or clipped to the needle hub. A small current is induced by the coil, as it moves within the changing electromagnetic field. This changing current reports three dimensional position coordinates and trajectory. Coregistration or matching of modalities is accomplished by placing fiducials or a fiducial patch on the skin near the field of interest. The coordinates are semiautomatically matched between the image and the fiducial, then a virtual image of the needle position is superimposed upon prior CT, MRI, and/or PET [2].

A composite fusion image can be displayed blending (or displaying side by side) two or more modalities along with the needle position. The accuracy and clinical impact of EM tracking systems for biopsy and ablation have been described in early reports [3, 4]. RF ablation in a patient with renal insufficiency was facilitated with EM tracking and multimodality fusion imaging that included real-time ultrasound with contrast as well as preprocedural unenhanced CT and MR. Using this approach, iodinated and gadolinium contrasts were not used for the ablation, and thus risks of renal damage (from iodine) and nephrogenic systemic fibrosis (from gadolinium) were minimized. 

## 2. Case Report 

**Figure 1:**
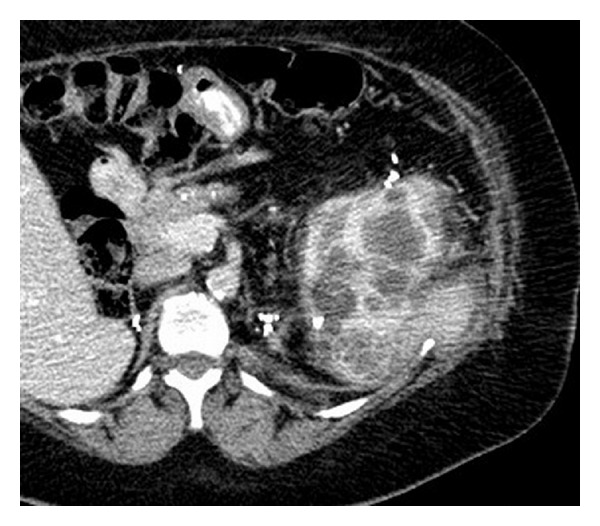
Preprocedure enhanced CT shows a mixed solid and cystic enhancing posterior renal tumor (6 o'clock in kidney).

A 53-year-old-female patient with a history of Von Hippel Lindau disease, multiple prior complex surgeries complicated by massive blood loss and transfusions, prior renal ablations, and recurrent renal tumors presented with a growing left, posterior, midpole, exophytic, 3 cm renal tumor, declining renal function, and renal insufficiency, with creatinine = 2.0 mg/dL. The patient gave a written informed consent for a clinical trial for EM tracking and ablation, approved by the institutional investigational review board. Noncontrast CT scan did not adequately visualize the tumor (Figure 4(c)), and contrast was not desirable due to renal insufficiency. MR (Philips 3.0T Achieva scanner, Philips Medical Systems, Best, The Netherlands) with gadolinium-DTPA contrast (Magnevist; Berlex Laboratories, Wayne, NJ, USA) and preprocedure enhanced CT showed the tumor margins well (Figure 1). 

**Figure 2:**
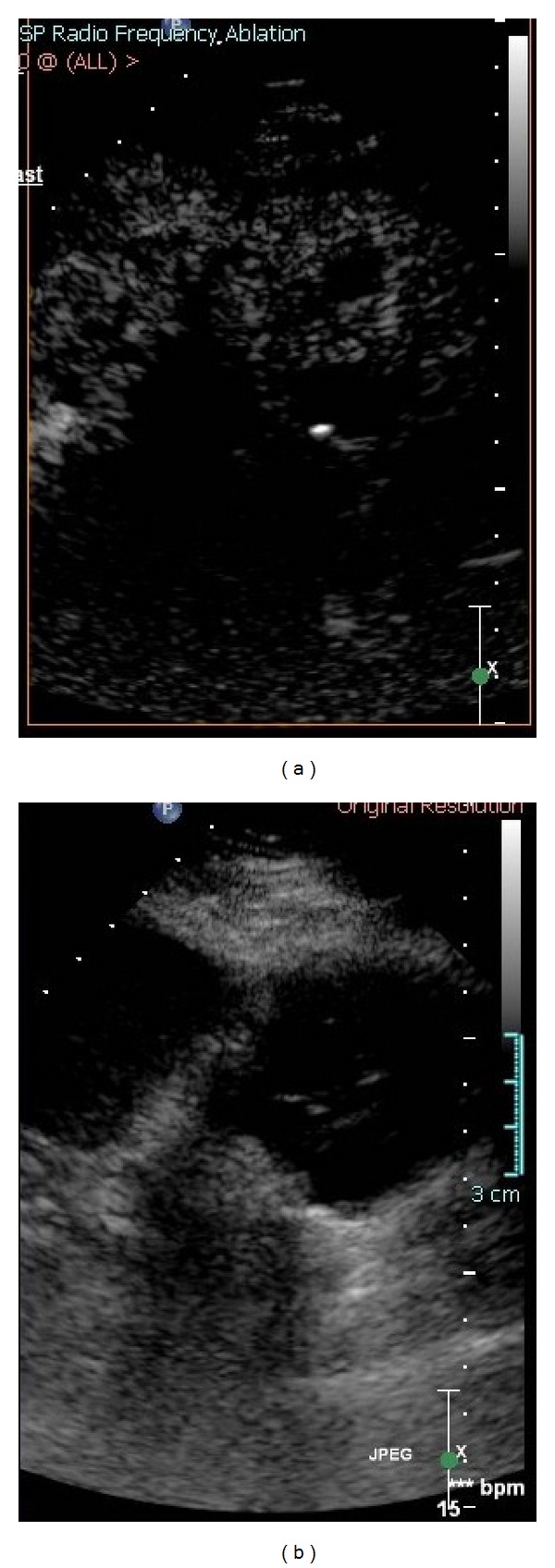
Ultrasound contrast image and grey scale ultrasound before ablation show the perfusion of solid mass (a) and hypoechoic mixed cystic and solid mass (b).

An EM tracking system and field generator were used (Figure 2) (Northern Digital Inc., Waterloo, ON, USA) interfaced with custom registration and display software (Philips Research, Briarcliff, NY, USA) and a commercially available tracked 22 G stylet inside a standard 19 G outer biopsy guider needle stylet (Philips Healthcare, Toronto, ON, USA). Five fiducial markers were placed near quadrant of interest. Ventilation was interrupted for CT imaging and for navigation. A preprocedural CT scan (MX 8000, Philips Medical Systems, Cleveland, OH, USA) was obtained during the interruption of ventilation, with 3 mm thick sections, 1.5 mm overlap. The skin fiducials were identified manually on the CT. The patient was supine for the planning MR and decubitus during CT and throughout the ablation procedure. Therefore, registration rotated the prior MR to match the preprocedural CT scan, and the target tumor was defined on the fusion images. 

The skin entry site was estimated using a radio-opaque grid (E-Z-EM Inc., Lake Success, NY, USA). The guider needle was inserted while the tracking software displayed the virtual needle position and trajectory, along with the target point, on the MR-CT composite image. Another CT scan was then taken to confirm the actual location of the needle, which was later compared to the displayed location, using previously described techniques [2–4]. This target to registration error was 1.5 mm. The root mean square registration error of image to actual fiducials was 1.7 mm.

To better delineate the tumor margin, as well as the postablation devascularized zone, US contrast was administered (Definity, Bristol Myers Squibb, N. Billerica, MA, USA) in 2 mL boluses (total 4 mL) during needle placement (Figure 2) as well as after RFA to confirm target and to confirm treatment zone, respectively. Similar to the tracked needle, the ultrasound transducer also had sensor coils attached, which allowed the ultrasound image to be tracked and referenced to the needle and the preprocedural CT and MRI images (Figure 4).

**Figure 3:**
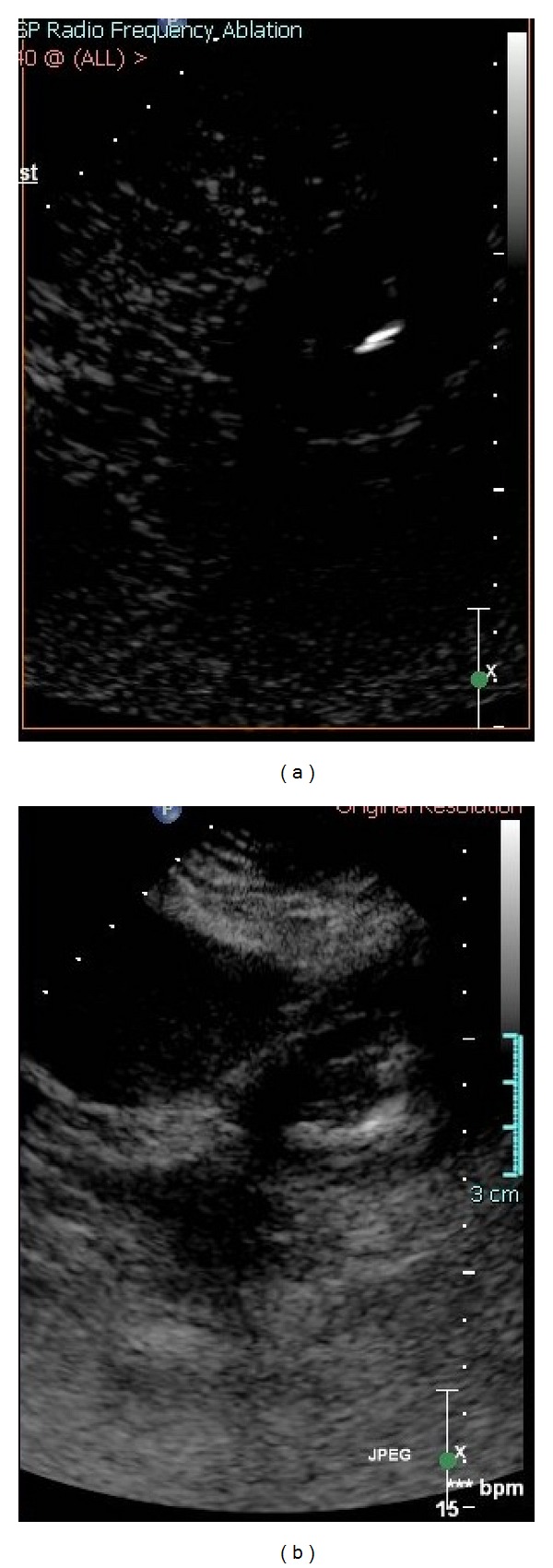
Ultrasound contrast image and grey scale ultrasound show the lack of the perfusion of solid mass with central needle (a) and now increased echoes in tumor from ablation (b).

**Figure 4:**
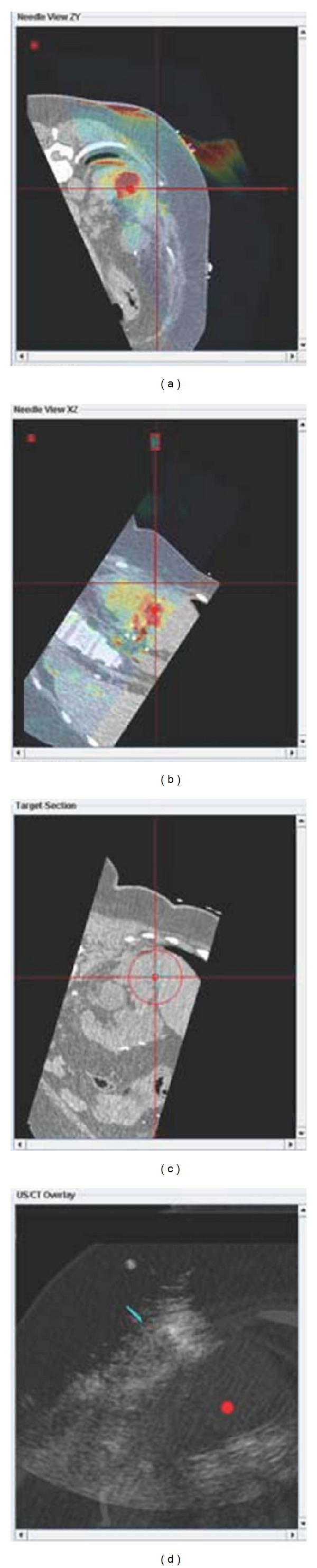
Fusion images combine procedural CT with MRI ((a) and (b) color images) and ultrasound (d) to display tumor target (red sphere (a) and (b) and red dot (d)). The tumor target is displayed in relation to the tracked needle location (cross hairs).

**Figure 5:**
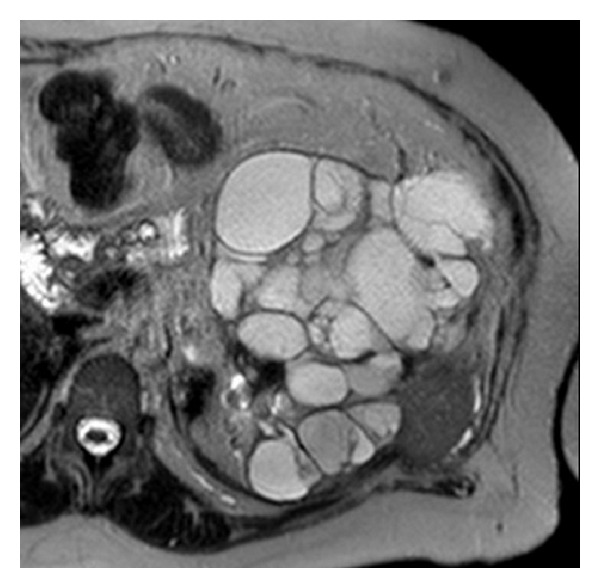
T2 weighted MRI 5 years 1 month after ablation shows total disappearance of solid lesion.

A single water cooled 17 G needle was placed using tandem technique with a 12-minute ablation, with needle position verified with fusion and ultrasound (Figure 4). Ultrasound contrast then confirmed residual tumor blood flow and viability; so a cluster needle (Cool-tip, Covidien, Boulder, CO, USA) was then introduced by similar technique. After-ablation, US contrast was not seen to perfuse the tumor, suggesting complete treatment (Figure 3). Postablation creatinine actually went down from 2.0 to 1.8 mg/dL. No tumor recurrence was seen at 5-year 1-month postablation MRI (Figure 5).

## 3. Discussion

With the increasing reliance of nephron-sparing techniques in the management of renal cancer, RFA has become a widely accepted form of treatment with even less damage to renal function than surgery [1, 5]. Also, renal tumors may only be seen on specific CT phases of the contrast bolus, or on specific sequences of MRI. This spatial information alone does not guarantee accurate nephron-sparing treatment, unless it is used in combination with fusion. Ideal visualization may improve accuracy for needle-based ablation procedures. 

The needle was guided with a CT-MR composite image overlaid with a real-time US. When using standard tools alone (and no EM tracking) in cases with limited tumor conspicuity, the physician mentally “registers” the information from offline preprocedure modalities onto the “blind” guidance modality using estimates of shared anatomical landmarks. This conventional method is not standardized and highly prone to human error inherent to such estimates. In this case, we electronically superimposed the MR target, well delineated by gadolinium enhancement, upon the procedural CT and US images, allowing the adequate visualization of the tumor without using nephrotoxic CT contrast (Figures 2–4). Limitations and sources of error for fusion imaging with electromagnetic tracking include nearby metal interference, limited working space, rigid registration, organ motion, and respiratory motion [2, 3].

The combination of US with CT and MR can make use of all three complementary modalities: US is an inexpensive and readily available method of obtaining real-time imaging feedback without ionizing radiation. US contrast adds information on tumor perfusion and viability. MR can localize soft tissue tumors often better than US or CT. However, US is often obscured by microbubbles released during heating. Multiplanar image reconstruction and real-time position feedback can facilitate needle placement. Although speculative, this could potentially reduce procedure time, decrease radiation exposure, reduce the need for multiple confirmation CTs, and may even avoid multiple needle insertions [6, 7]. Accuracy may be especially important for nephron-sparing procedures.
